# *Helicobacter pylori* virulence genes of minor ethnic groups in North Thailand

**DOI:** 10.1186/s13099-017-0205-x

**Published:** 2017-10-11

**Authors:** Phawinee Subsomwong, Muhammad Miftahussurur, Ratha-korn Vilaichone, Thawee Ratanachu-ek, Rumiko Suzuki, Junko Akada, Tomohisa Uchida, Varocha Mahachai, Yoshio Yamaoka

**Affiliations:** 1 Department of Environment and Preventive Medicine, Faculty of Medicine, Oita University, 1-1 Idaigaoka, Hasama-machi, Yufu, Oita 879-5593 Japan; 2 Gastroentero-Hepatology Division, Department of Internal Medicine, Faculty of Medicine-Institute of Tropical Disease, Universitas Airlangga, Surabaya, 60115 Indonesia; 3 Gastroenterology Unit, Department of Medicine, Thammasat University Hospital, Amphoe Khlong Luang, Pathum Thani 12120 Thailand; 4 Department of Surgery, Rajavithi Hospital, Bangkok, 10400 Thailand; 5 Department of Molecular Pathology, Faculty of Medicine, Oita University, Hasama-machi, Yufu, Oita 879-5593 Japan; 6 GI and Liver Center, Bangkok Medical Center, Bangkok, 10310 Thailand; 7 Department of Medicine-Gastroenterology, Baylor College of Medicine, Houston, TX 77030 USA

**Keywords:** *Helicobacter pylori*, Virulence factors, Minor ethnics, North Thailand, Human migration

## Abstract

**Background:**

There are few studies analyzed concurrently the prevalence and genotypes of *Helicobacter pylori* infection with the ancestor origins from different ethnics, especially with including minority groups. We recruited a total of 289 patients in MaeSot, Thailand (154 Thai, 14 Thai-Chinese, 29 Karen and 92 Hmong ethnics). The virulence genes and genealogy of the strains were determined by PCR-based sequencing.

**Results:**

Based on culture and histology/immunohistochemistry, the prevalence of *H. pylori* infection was 54.5% (158/289). Among 152 isolates cultured, the East-Asian-type *cagA* was predominant genotype among strains from Hmong, Thai-Chinese and Thai (96.0% [48/50], 85.7% [6/7] and 62.7% [47/75], respectively), whilst majority of strains from Karen had Western-type *cagA* (73.3% [11/15]). Patients infected with the East-Asian-type *cagA* strains had significantly higher activity and intestinal metaplasia in the antrum and activity in the corpus than those with Western-type *cagA* (*P* = 0.024, 0.006 and 0.005, respectively). The multilocus sequencing typing analysis discriminated that most strains from Hmong and Thai-Chinese belonged to hspEAsia (92.0 and 85.7%, respectively), whereas strains from Karen predominantly possessed hpAsia2 (86.7%) and strains from Thai were classified into hspEAsia (45.2%) and hpAsia2 (31.1%).

**Conclusions:**

*Helicobacter pylori* genotypes were relatively different among ethnic groups in Thailand and were associated with the source of ancestor even living in a small rural town. Caution and careful check-up are required especially on Hmong ethnic associated with high prevalence of virulence genotypes of *H. pylori*.

**Electronic supplementary material:**

The online version of this article (doi:10.1186/s13099-017-0205-x) contains supplementary material, which is available to authorized users.

## Background

*Helicobacter pylori* is a microaerophilic spiral shaped bacteria colonizing gastric mucosa on approximately half of the global population and is related with severe gastroduodenal diseases including peptic ulcers and gastric cancer [1]. The incidence of gastric cancer is highest in East Asia, whereas it is relatively low in Africa and South Asia although the prevalence of *H. pylori* infection was highest in these area [2]. Gastric cancer incidence has a tendency decreasing from North to South even within East Asia. This phenomenon could be explained partly by diversity of *H. pylori* virulence [3]. For example, most of *H. pylori* isolates from East Asia are CagA-positive, while approximately 20 to 40% of isolates from Europe and Africa are CagA-negative [4]. In addition, the classification of CagA into East-Asian-type and Western-type based on the differences of the repeat sequence (Glu-Pro-Ile-Tyr-Ala [EPIYA] segments) are likely associated with the incidence of gastric cancer [5, 6] as well as a geographical distribution [7]. The *vacA* m1 type strains are common in areas of Northeast Asia, i.e., Japan and South Korea, whereas m2 type strains are predominant in areas of Southeast Asia, i.e., Taiwan and Vietnam [8]. It will be interesting to analyze other virulence factors as geographical diversity marker such as duodenal ulcer promoting gene (*dupA*), blood group antigen-binding adhesin (*babA*), induced by contact with epithelium *(iceA*) and *jhp0562* and β-*galT*-*(jhp0563)* which were also reported to have association with severe clinical outcomes [9]. However, to our knowledge, there are no detailed studies investigating the status of these virulence genes in relation to the geographic/ethnic differences.

Previous study revealed a benefit utilization of multilocus sequence typing (MLST) analysis of *H. pylori* which provided human population structure information in detail rather than other methods such as human microsatellite or mitochondrial DNA [10]. MLST assesed seven *H. pylori* populations based on seven housekeeping genes (*atpA, efp, mutY, ppa, trpC, ureI,* and *yphC*) including hpAfrica1, hpAfrica2, hpNEAfrica, hpEurope, hpEastAsia, hpAsia2, and hpSahul that could forecasting human migrations pattern [11, 12]. The hpAfrica1, hpAfrica2 and hpNEAfrica strains are predominant in isolates from Africa, hpEurope strains are mainly isolated from ethnic Europeans, hpEastAsia strains are common in *H. pylori* from East Asia, hpAsia2 strains are common in *H. pylori* from South, Southeast, and Central Asia, and hpSahul strains are mainly isolated from aborigines of Australia and highlanders in New Guinea [3].

Thailand is Southeast Asian country bordering with Myanmar in the North and West, Laos in the North and North-East, Cambodia in the East and with Malaysia in the South. Although Thai and Chinese are the major ethnic groups, Thailand is a multi-ethnic country which also consists of numerous minor tribes living in the mountain area of the North Thailand. Especially, Maesot, a district on Tak province in North region is a unique area with high culturally diverse due to several ethnic groups such as Thai, Burmese and minor ethnic people with unknown origin (Hmong and Karen) living together in the small town (120,569 people in 2008, Statistical yearbook Thailand 2013, http://web.nso.go.th). Therefore MaeSot is an ideal area for focusing the roles of *H. pyori* virulence factors among different ethnic groups as well as for mapping human migration patterns. In this study, we therefore aimed to clarify two important goals; (1) *H. pylori* infection and the virulence genotypes, and (2) the origin of ethnics by using *H. pylori* as a tracking tool. We hypothesized that different ethnics had different genes characteristics of *H. pylori*, which would be associated with different ancestry.

## Methods

### Study population

We conducted a community-based endoscopic survey at Ban Chedi Ko School, Maesot city, Tak province during November 11–12, 2013. All the endoscopy equipments were brought from Bangkok and were set up at the gymnastic hall of the school. The project was announced by the local hospital officers 1 month before the survey. Four major ethnic groups including Thai, Thai-Chinese, Karen and Hmong who living in the same community were participated during the survey. Thai-Chinese were defined as Thai citizens who were the offspring of mixed marriages [13]. Gastric biopsy specimens were collected from each patient from the antrum and corpus of the stomach; two samples from the lesser curvature of the antrum approximately 3 cm from pyloric ring, and one sample from the greater curvature of the corpus. Two specimens from the antrum were used for *H. pylori* culture and histological examination. One specimen from the corpus was used for histological examination. The biopsy specimens for culture were immediately placed at − 20 °C, and stored at − 80 °C within a day of collection until used for culture. Peptic ulcers were diagnosed by endoscopic examinations. The normal gastric mucosa was defined as the absence of any activity and inflammation in both the antrum and corpus by histological examination. Blood samples were collected from all participants on the same day of endoscopy for measuring serum anti-*H. pylori* antibody.

Written informed consent was obtained from all participants, and the study protocol was approved by Human Research Ethnics Committee of Faculty of Medicine, Thammasat University (Pathum Thani, Thailand) and Oita University Faculty of Medicine (Yufu, Japan).

### Diagnosis of *H. pylori* infection and gastritis stage determination

The *H. pylori* infection status was diagnosed by serum anti-*H. pylori* antibody by enzyme-linked immunosorbent assay kit (Eiken Co., Ltd., Tokyo, Japan), culture, histology including immunohistochemistry (IHC). For *H. pylori* culture, the biopsy specimen was homogenized in normal saline and was inoculated onto *H. pylori* selective media (Nissui Pharmaceutical Co., Ltd., Tokyo, Japan). The plates were incubated for up to 7 days at 37 °C under microaerophilic conditions (10% O_2_, 5% CO_2_, and 85% N_2_). The *H. pylori* like colony were sub-cultured onto Mueller–Hinton II Agar medium (Becton–Dickinson, Sparks, MD, USA) supplemented with 7% horse blood (Nippon Bio-test, Tokyo, Japan) without antibiotics. *H. pylori* was identified on the basis of colony morphology, gram staining and positive reactions for oxidase, catalase, and urease. Isolated strains were stored at − 80 °C in Brucella Broth (Becton–Dickinson, Sparks, MD, USA) containing 10% dimethyl sulfoxide and 10% horse serum.

All biopsy specimens for histological testing were fixed in 10% buffered formalin and embedded in paraffin. Serial sections were stained with hematoxylin and eosin as well as May–Giemsa stains. The degree of inflammation (monocyte infiltration), activity (neutrophil infiltration), atrophy, intestinal metaplasia, and bacterial density were classified into four grades according to the updated Sydney system: 0, ‘normal’; 1, ‘mild’; 2, ‘moderate’; and 3, ‘marked’ [14]. Samples with grade 1 or more atrophy were considered atrophy-positive [15]. In addition, gastritis stage was assessed based on topographic locations (antrum and corpus), according to the Operative Link on Gastritis Assessment (OLGA) system [16].

IHC was performed as previously described [17]. Briefly, after antigen retrieval and inactivation of endogenous peroxidase activity, tissue sections were incubated with anti-*H. pylori* antibody (DAKO, Glostrup, Denmark), anti-CagA antibody (b-300 Santa Cruz, CA, USA) or anti-East-Asian-type CagA-specific antibody (α-EAS Ab) diluted 1:2000 with diluting solution (DAKO) overnight at 4 °C. The α-EAS Ab was immunoreactivity with only the East-Asian-type CagA, and not with the Western-type-CagA [18]. IHC using the α-EAS Ab was proven to be useful tool for typing East-Asian-type CagA immunohistochemically in Japan [17], Vietnam and Thailand [19]. After washing, the sections were incubated with biotinylated goat anti-rabbit or anti-rat IgG (Nichirei Co., Tokyo, Japan), followed by incubation with a solution of avidin-conjugated horseradish peroxidase (Vectastain Elite ABC kit; Vector Laboratories Inc., Burlingame, CA, USA). Peroxidase activity was detected using H_2_O_2_/diaminobenzidine substrate solution. *H. pylori* were identified by Giemsa staining and positively immunostained with anti-*H. pylori* antibody. The bacterial density scores evaluated by the updated Sydney system equal or greater than grade 1 were considered positive for *H. pylori* infection. *H. pylori* infection was clarified when histology confirmed by IHC and/or culture tests showed positive.

### *H. pylori* genotyping

DNA was extracted from *H. pylori* culture using the QIAamp DNA Mini Kit (QIAGEN, Valencia, CA, USA) according to the manufacturer’s directions. The DNA was kept at − 20 °C until used for genotyping study. The *cagA* genotypes (positivity, EPIYA repeat region and pre-EPIYA) were determined by polymerase chain reaction (PCR) amplification-based direct sequencing using the ABI 3100 Genetic Analyzer DNA Sequencer. The *vacA* genotypes (s1 or s2, m1 or m2, i1 or i2, d1 or d2 and c1 or c2 regions), *iceA* genotype (*iceA1* or *iceA2*), and the presence of *dupA, babA, jhp0562*, and *β*-*(1,3)galT* genes were determined based on PCR product size (Additional file 1: Tables S1.) as described previously [20–27] by Microchip Electrophoresis system for DNA/RNA Analysis MCE^®^ -202 Multina (Shimadzu, Japan).

### Population structure analysis of *H. pylori* strains

The *H. pylori* population was constructed by MLST sequence datasets comprised of seven housekeeping genes (*atpA, efp, mutY, ppa, trpC ureI,* and *yphC*) from 430 strains with different genotype obtained from the pubMLST data base (http://pubmlst.org/). These sequence datasets were integrated with Maesot sequence data. A neighbor-joining tree was constructed based on the sequence alignment using MEGA v.6.06. We analyzed bacterial population structure using STRUCTURE (v.2.3.2) software. Markov chain Monte Carlo simulations of STRUCTURE were run in the non admixture model with burn-in of 20,000 followed by 30,000 iterations for each run. To run STRUCTURE, a hypothetical number of bacterial populations, K, must be input. We set K as 8–15 and performed five runs for each K.

### Data analysis

Data were analyzed by using SPSS, version 16 (SPSS Inc., Chicago, IL, USA). The discrete variables were tested by using Chi square test while continuous variables were tested by Mann–Whitney *U* test and *t* test. The *P*- value < 0.05 was considered statistically significant.

### Nucleotide sequencing

Nucleotide sequence data of *cagA*-positive strains are available under the DDBJ Accession Numbers LC092222 to LC092367. The sequencing data for the seven housekeeping genes of the 89 strains are available under the DDBJ Accession Numbers LC092368 to LC092829 and LC271796 to LC271956.

## Results

### Prevalence of *H. pylori* in Maesot

We performed endoscopy for 295 patients; six were excluded due to the remaining foods in the stomach. Finally, a total population were 289 patients (217 females and 72 males, mean age, 47.0 ± 14.9 years; range, 9–89 years) consisted of 38 aged ≤ 29 years old, 59 aged 30–39 years old, 73 aged 40–49 years old, 53 aged 50–59 years old, 45 aged 60–69 years old and 21 aged ≥ 70 years old. They were consisted of 154 Thai, 14 Thai-Chinese, 29 Karen and 92 Hmong ethnics.

**Fig. 1:**
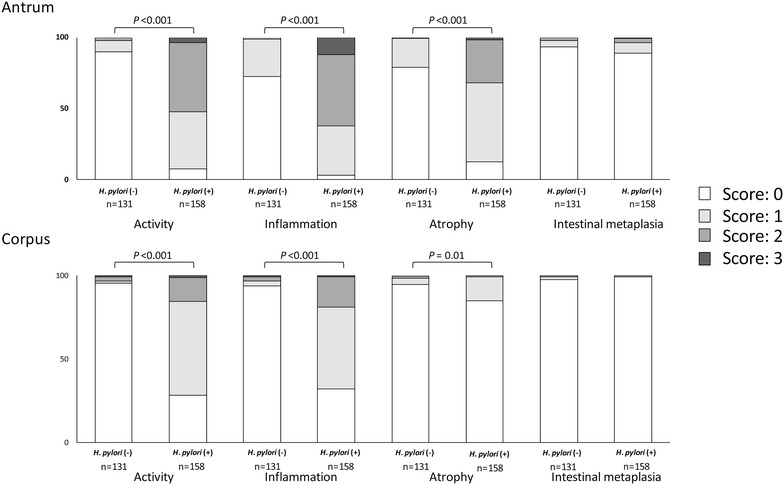
Histological score according to *H. pylori* infection status. The *H. pylori* positive (n = 158) induced activity, inflammation and atrophy in both of the antrum and corpus were significantly higher than *H. pylori* negative (n = 131, *P* < 0.05, Mann–Whitney U test)

The prevalence of *H. pylori* infection by three diagnosis methods is shown in Table 1. *H. pylori* antibody detection had the highest positive rate (54.7%), followed by histology confirmed by IHC (54.0%) and culture (52.6%). When patients were considered to be *H. pylori* positive in case at least one test showed positive, the prevalence of *H. pylori* infection was 60.0%. Fifteen patients (15/158, 9.5%) had positive results in serology test only, but negative in other tests, thus we predicted as a past *H. pylori* infection. When we clarified *H. pylori*-positive if histology confirmed by IHC and/or culture yielded positive results, the prevalence was 54.5%. We used this criteria in the subsequent analyses. Hmong ethnic had the highest prevalence of *H. pylori* infection (59.8%, 55/92), followed by Karen (55.2%, 16/29), Thai (51.9%, 80/154) and Thai-Chinese ethnic (50.0%, 7/14). The histological severity according to *H. pylori* status was shown in Fig. 1. As expected, patients infected with *H. pylori* had significantly higher histological scores both in the antrum and corpus than uninfected patients even after adjusted with age, sex and ethnic groups [OR (Odd ratio):113.90 and 88.49 for activity; 30.64 and 51.97 for inflammation; 37.90 and 3.04 for atrophy in the antrum and corpus, respectively].

**Table 1:** Prevalence of *H. pylori* infection by diagnosis methods and ethnicity

	Age (years)						
	≤ 29	30–39	40–49	50–59	60–69	≥ 70	Total
N	38	59	73	53	45	21	289
Culture (%)	13 (34.2)	36 (61.0)	38 (52.1)	32 (60.4)	25 (55.6)	8 (38.1)	152 (52.6)
Histology confirmed by IHC (%)	13 (34.2)	37 (62.7)	40 (54.8)	33 (62.3)	25 (55.6)	8 (38.1)	156 (54.0)
*H. pylori* antibody by ELISA (%)	17 (44.7)	38 (64.4)	40 (54.8)	31 (58.5)	25 (55.6)	7 (33.3)	158 (54.7)
Past infection^a^ (%)	5 (13.2)	2 (3.4)	4 (5.5)	2 (3.8)	1 (2.2)	1 (4.8)	15 (5.2)
At least one test-positive (%)	18 (47.4)	40 (67.8)	44 (60.3)	35 (66.0)	27 (60.0)	9 (42.9)	173 (60.0)
Histology confirmed by IHC and/or culture-positive (%)	13 (34.2)	38 (64.4)	40 (54.8)	33 (62.3)	26 (57.8)	8 (38.1)	158 (54.5)

*IHC* immunohistochemistry

^a^Positive results in serology test only, but negative in other tests

### *vacA* and *cagA* genotypes

A total of 152 strains were successfully cultured; 108 were isolated from females (age range 18–89 years; mean age 46.6 years) and 44 were from males (age range 12–88 years; mean age 49.5 years). Among the cultured strains; 77 were isolated from Thai subjects, 53 from Hmong, 15 from Karen and 7 from Thai-Chinese. We found *vacA* s1, m1, i1, d1 and c1 were the predominant genotypes in Thai, Hmong and Karen ethnics, whereas Thai-Chinese had high proportion of *vacA* s1, m2, i1, d1 and c1 and 2 (Table 2). There were significant differences of ethnic groups with *vacA i* type (*P* < 0.001).

**Table 2:** The distribution of *H. pylori* virulence factors by ethnicity

	Ethnicity (number %)				
	Thai	Hmong	Karen	Thai-Chinese	Total
Cultured (N)	77	53	15	7	152
*vacA* s1	76 (98.7)	53 (100.0)	15 (100.0)	7 (100.0)	151 (99.3)
m1	45 (58.4)	31 (58.5)	13 (86.7)	2 (28.6)	91 (59.9)
i1	75 (97.4)	53 (100.0)	15 (100.0)	5 (71.4)	148 (97.4)
d1	74 (96.1)	53 (100.0)	15 (100.0)	6 (85.7)	148 (97.4)
c1	44 (57.1)	23 (43.4)	12 (80.0)	1 (14.3)	80 (52.6)
*cagA* positive	75 (97.4)	50 (94.3)	15 (100.0)	7 (100.0)	147 (96.7)
East-Asian-type-*cagA*	47 (62.7)	48 (96.0)	4 (26.7)	6 (85.7)	105 (71.4)
Western-type-*cagA*	27 (36.0)	2 (4.0)	11 (73.3)	1 (14.3)	41 (27.9)
Predominant upstream EPIYA motif	74	50	15	7	146
No-deletion	30 (40.5)	2 (4.0)	11 (73.3)	1 (14.3)	44 (30.0)
18-bp deletion	25 (33.8)	27 (54.0)	4 (26.7)	2 (28.6)	58 (40.0)
39-bp deletion	19 (25.7)	21 (42.0)	0 (0.0)	4 (57.1)	44 (30.0)
*dupA* negative	60 (77.9)	39 (73.6)	15 (100.0)	4 (57.1)	118 (77.6)
*babA* positive	72 (93.5)	52 (98.1)	15 (100.0)	6 (85.7)	145 (95.4)
*iceA1* genotype	59 (76.6)	48 (96.0)	8 (53.3)	6 (85.7)	121 (79.6)
*Jhp0562* positive, *β*-*(1,3)gal T* negative	42 (54.5)	40 (75.5)	8 (53.3)	6 (85.7)	96 (63.2)

We found 147 strains were *cagA*-positive (96.7%) and only five strains (two from Thai and three from Hmong ethnic) were confirmed to be *cagA*-negative by *cag* PAI empty site PCR [28]. Among the *cagA*-positive strains, the East-Asian-type *cagA* strains were 71.4% (ABD and ABBD), Western-type-*cagA* strains were 27.9% (AB, ABC, ABCC, B, BC and BCC) and one strain could not determine the genotype according to unclear sequencing result. The high proportion of East-Asian-type *cagA* was found among Hmong, Thai-Chinese and Thai strains (96.0, 85.7 and 62.7%), in contrast, Western-type *cagA* was predominant in Karen strains (73.3%) and Thai strains (36.0%).

The analysis of 300 bp upstream of the first EPIYA motif (pre-EPIYA) in Maesot strains revealed that East-Asian-type *cagA* had 18-bp (55.2%, 58/105) or 39-bp deletion types (41.9%, 44/105) which is reported to be typically observed in Vietnamese and East Asian countries strains, respectively [24] and only three strains (2.9%) had no deletion. While 93.2% (41/44) of no deletion type had Western-type *cagA* and only 6.8% (3/44) was East-Asian-type *cagA.* No deletion type is reported to be typically observed in strains from Western countries. Concordance with *cagA* genotype, no deletion type was predominant in Karen and Thai strains (73.3 and 40.5%, respectively), whereas the majority of Thai-Chinese and Hmong strains had 39-bp deletion (57.1%) and 18-bp deletion (54.0%).

### CagA and α-EAS Ab

We performed IHC to detect immunoreactivity with CagA and α-EAS Ab. Overall, 146 of 147 samples were positive for *H. pylori* anti-CagA Ab. In line with genotyping using PCR; among CagA-positive cases, Thai-Chinese (5/6, 83.3%), Hmong (39/53, 73.6%) and Thai (30/48, 62.5%) had higher proportion for positive immunoreactivity to α-EAS Ab than Karen (1/4, 25.0%). As expected, all 41 samples infected with Western-type *cagA* strains showed negative immunoreactivity with α-EAS Ab. However, only 75 out of 105 samples (71.4%) infected with East-Asian-type *cagA* strains were positive for α-EAS Ab including one strain which could not define the genotype. Using PCR-based sequencing for *cagA* as the gold standard, the sensitive, specificity, negative predictive value, and positive predictive value of the α-EAS Ab were 70.8, 97.8, 59.2 and 98.7%, respectively. These results was much lower sensitivity than those in previous study [19]. Thus, we further analyzed all the *cagA*-positive strains for the corresponding area of target epitope sequences of α-EAS Ab to obtain an argument. The sequences were consist of 18 amino acid; AINRKIDRINKIASAGKG in EPIYA segment D, based on the sequences of Japanese strains [29]. Among 75 East-Asian-type *cagA* strains which were positive by α-EAS Ab, 73 (97.3%) had identical with the target α-EAS Ab sequences and remaining two strains had two amino acid sequences different from the epitope sequence target. On the other hand, between 30 East-Asian-type *cagA* strains that were negative by α-EAS Ab, only eight strains had identical with the target site, while remaining 22 strains had different amino acid at the epitope sequence (3–14 amino acid difference). Importantly, those six strains could not detected with anti-CagA antibody indicated a false positive of the kit.

### Gastric mucosal status, ethnics and virulence genes

**Fig. 2:**
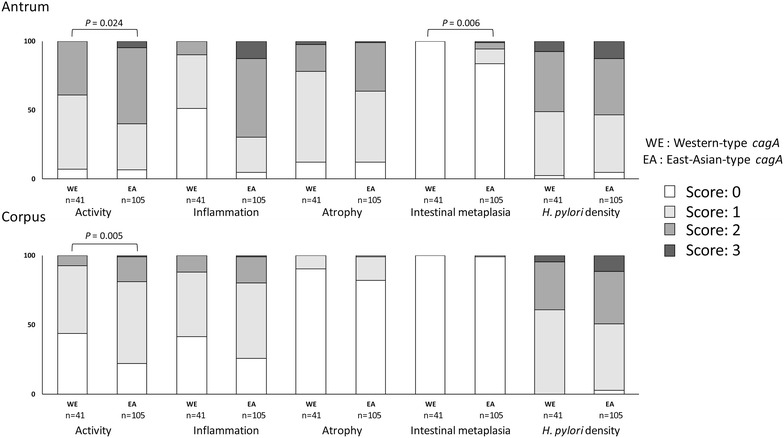
Histological score according to genotype of *cagA. H. pylori* with East-Asian-type *cagA* (n = 105) had significant higher level of activity in antrum and corpus and intestinal metaplasia in antrum than *H. pylori* with Western-type *cagA* genotype (n = 41, *P* < 0.05, Mann–Whitney U test)

The patients infected with East-Asian-type *cagA* strains showed significantly higher activity and intestinal metaplasia scores in the antrum (*P* = 0.024 and *P* = 0.006), and activity scores in the corpus (*P* = 0.005) than those with Western-type *cagA* strains (Fig. 2); however there was no statistical difference in OLGA score. Similar results were found in subanalysis of *cagA* genotype in both Thai and Karen ethnics (Additional file 2: Figures S1 and Additional file 3: Figure S2). Since the number of Western-type-*cagA* strains in Hmong and Thai-Chinese ethnics were too small (two and one, respectively), same analysis could not be performed. The patients infected with pre-EPIYA 18-bp deletion type strains had significantly higher activity and intestinal metaplasia scores in the antrum, and activity score in the corpus than those with no deletion type strains (*P* = 0.01, *P* = 0.009 and *P* = 0.002, respectively) (Additional file 1: Table S2). Interestingly, patients infected with 18-bp deletion type strains had significantly higher activity score in the corpus than those with 39-bp deletion types strains (*P* = 0.01). Subethnic analysis found that Thai subjects infected with pre-EPIYA 18-bp deletion type strains had significant higher activity, atrophy in the antrum, activity in the corpus and OLGA score than those with no deletion type and 39-bp deletion type strains (*P* < 0.001, *P* < 0.001, *P* = 0.018, *P* < 0.001 and *P* = 0.045, *P* = 0.049, *P* = 0.01 and *P* = 0.049, respectively). Similar results also were found in Karen ethnic subanalysis. Thai subjects infected with 39-bp deletion type strains had significantly higher intestinal metaplasia score in the antrum than those with no deletion type strains (*P* = 0.046). In contrast, the histological score by *vacA* genotype was not significantly different between sub-genotypes (*P* > 0.05, Additional file 1:Tables S3).

### Others virulence genes genotypes and gastric mucosal status

We examined the presence/absence of several other virulence genes; *dupA, babA, jhp0562*, and *β*-*(1,3)galT* and the *iceA* genotype (*iceA1* or *iceA2*) (Table 2)*. H. pylori* with *babA* positive, *iceA1* type*, dupA* negative and *jhp0562* positive was majority in Maesot strains (95.4, 79.6, 77.6 and 63.2%, respectively). We found high proportion of *dupA*-negative strains in Karen, Thai, Hmong and Thai-Chinese (100.0, 77.9, 73.6 and 57.1%, respectively, *P* = 0.089). The distribution of *iceA*1 genotype was high in Hmong, Thai-Chinese, Thai and Karen (96.0, 85.7, 76.6 and 53.3%, respectively, *P* = 0.055). Majority of Thai-Chinese strains had *jhp0562* positive (85.7%), followed by Hmong, Thai and Karen (75.5, 54.5 and 53.3%, respectively, *P* = 0.077). There was no significant difference of *dupA*, *babA*, *iceA* and *jhp0562*/*β*- *(1, 3)galT* status with gastric mucosal status (*P* > 0.05).

### *H. pylori* population structure

We selected all strains with specific *cagA* genotypes from each ethnic group if the number of cultured *H. pylori* was equal or less than 20, whereas randomly selected 20 strains with specific *cagA* genotypes if the number was more than 20. As a result, we examined 89 strains including 42 strains cultured from Thai ethnic (two strains with *cagA* negative, 20 strains with East-Asian-type and 20 strains with Western-type *cagA*), 25 strains from Hmong ethnics (three strains with *cagA* negative, 20 strains with East-Asian-type and two strains with Western-type *cagA*), 15 strains from Karen (4 East-Asian-type and 11 Western-type *cagA* strains) and seven strains from Thai-Chinese (6 East-Asian-type and 1 Western-type *cagA*). The MLST sequence data of strains from Maesot were integrated with 430 strains deposited in GenBank.

**Fig. 3:**
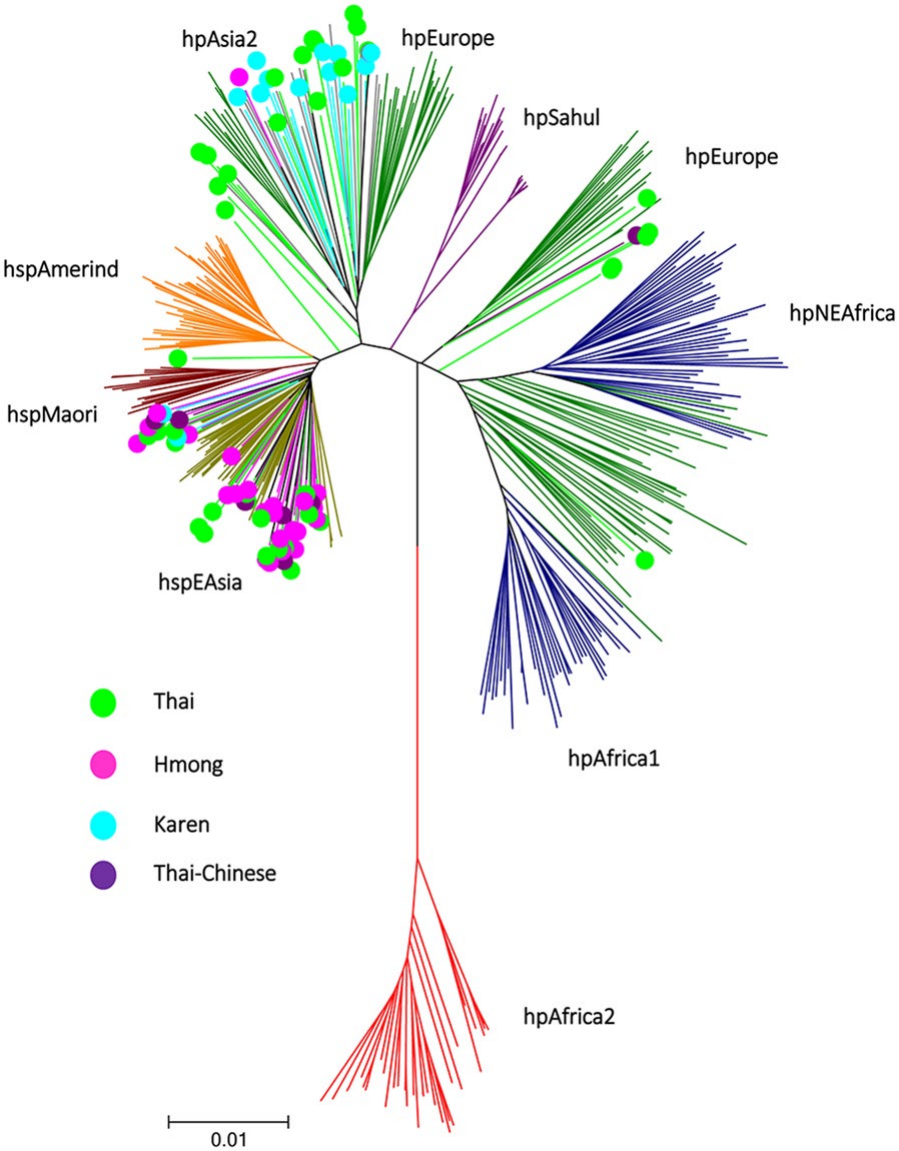
MLST phylogenetic tree of Maesot strains. *H. pylori* 89 strains (42 Thai, 25 Hmong, 15 Karen and 7 Thai-Chinese strains) and integrated with 430 strains that deposited in GenBank. The majority of the strains belonged to hspEAsia and hpAsia2

The phylogenetic tree is shown in Fig. 3 and Table 3. Maesot strains belonged to two major groups as hspEAsia (56.2%) and hpAsia2 (31.5%). The remains were hpEurope (5.6%) and undetermined (6.7%). The Hmong and Thai-Chinese strains had hspEAsia as major population type (92.0 and 85.7%, respectively), while majority of Karen strains had hpAsia (86.7%). Thai strains were categorized onto hspEAsia (45.2%), hpAsia2 (31.1%), hpEurope (9.5%) and undetermined type (14.3%). Most of East-Asian-type *cagA* genotype had hspEAsia (90.0%), while 73.5% of Western-type *cagA* genotype had hpAsia2 type.

**Table 3:** The distribution of *H. pylori* MLST in Maesot by ethnic group, *cagA* and *vacA* genotype

	*H. pylori* population (%)				Undetermined
	N	hspEAsia	hpAsia2	hpEurope	
Ethnic					
Thai	42	19 (45.2)	13 (31.1)	4 (9.5)	6 (14.3)
Hmong	25	23 (92.0)	2 (8.0)	0 (0.0)	0 (0.0)
Karen	15	2 (13.3)	13 (86.7)	0 (0.0)	0 (0.0)
Thai-Chinese	7	6 (85.7)	0 (0.0)	1 (14.3)	0 (0.0)
Total	89	50 (56.2)	28 (31.5)	5 (5.6)	6 (6.7)
*vacA*					
m1	60	26 (43.3)	26 (43.3)	3 (5.0)	5 (8.4)
m2	28	23 (82.2)	2 (7.1)	2 (7.1)	1 (3.6)
m1, 2	1	1 (100.0)	0 (0.0)	0 (0.0)	0 (0.0)
Total	89	50 (56.2)	28 (31.5)	5 (5.6)	6 (6.7)
*cagA*					
East-Asian-type	50	45 (90.0)	3 (6.0)	0 (0.0)	2 (4.0)
Western-type	34	2 (5.9)	25 (73.5)	4 (11.8)	3 (8.8)
Negative	5	3 (60.0)	0 (0.0)	1 (20.0)	1 (20.0)
Total	89	50 (56.2)	28 (31.5)	5 (5.6)	6 (6.7)

When we analyzed by STRUCTURE (Additional file 4: Figure S3.), we found that 90% (45/50) of hspEAsia population had East-Asian-type *cagA* and 89.3% (25/28) of hpAsia population had Western-type *cagA*. The combination of *H. pylori* population, *cagA* and *vacA* genotype is showed in Additional file 1: Table S4. The *H. pylori* with hspEAsia mostly harbored East-Asian-type *cagA* and *vacA* m1 or m2 (48.0 and 40.0%), while hpAsia2 mainly with Western-type *cagA* and *vacA* m1 (85.7%). Thai and Hmong strains with hspEAsia had East-Asian-type *cagA* and *vacA* m1 predominant (52.6 and 52.2%) while Thai-Chinese strains had *vacA* m2 (66.7%). Karen strain with hpAsia2 had Western-type *cagA* and *vacA* m1 predominant (76.9%) as well as Thai hpAsia2 strain (92.3%).

## Discussion

We revealed *H. pylori* infection in Maesot was 55.5%, an intermediate rate when compared to the previous reports about the prevalence of *H. pylori* infection in Thailand that showed in a big range from 17.5–88.5% [13, 18, 30–45]. To our knowledge, this is the first report about *H. pylori* prevalence in minor ethnics of Thailand. Interestingly, our results showed that both of Hmong and Karen ethnics who called as “minor mountain peoples” had a higher prevalence of *H. pylori* infection than predominant ethnics. Although environmental factors such as foods, habit and sanitary could influence the prevalence of *H. pylori*, our analysis showed that it is associated with the difference source of ancestry.

Totally, *H. pylori* with East-Asian-type *cagA* and 18-bp or 39-bp deletion types was the majority genotype in Maesot strains. Histological results confirmed that East-Asian-type *cagA* strains induced severer histological scores than those infected with Western-type *cagA*, concordance with the current consensus [46–48]. In contrast with our expectation, strains with pre-EPIYA 18-bp deletion also induced greater score in histology rather than other genotypes. In the current consensus 39-bp deletion types strains (mostly in East Asian countries) could induce severer histological scores than 18-bp deletion strains (predominant in Vietnam) since the fact that gastric cancer incidence in East Asian countries such as Japan, Korea and China are higher than that in Vietnam. It is suggested that pre-EPIYA region may only become marker of geographical diversity in each population rather than associated with clinical outcomes. Maesot strains also had virulence genotypes of *vacA* including s1, m1, i1, d1 and c1. Moreover, they also contained predominant of virulence genotype such as *babA* positive, *iceA1* type and *jhp0562* positive. Therefore, Maesot *H. pylori* theoretically have enough ability as an agent for inducing severe gastrointestinal diseases. However, Age standardize incidence rate (ASR) for gastric cancer in North Thailand (4.1, 5.4/100,000 in female and male) is still much lower than other countries (e.g., lower than Myanmar [11.2/100,000] or Vietnam [16.3/100,000]) suggesting host and environmental factors are dominant as denominator for clinical outcomes in Thailand rather than bacterial factors (data available from the International Agency for Research on Cancer; GLOBOCAN2012, http://globocan.iarc.fr/). Interestingly, we found high proportion of *dupA*-negative strains in Maesot that probably become as a specific marker for Maesot population as similar as in Indonesian population [49].

It is still questionable why the genotypes in Maesot strains were relatively consistent with minimal mixture between ethnics although living in the same area. Previously we found a mixed genotype in Thai-Chinese [13]. The virulence genes analysis showed that majority of Hmong, Thai-Chinese and Thai ethnics were infected with East-Asian-type *cagA* strains, whereas most of Karen strains were Western-type *cagA*. In addition, pre-EPIYA no deletion type, which is reported as the major type in Western population was also predominant in Karen strains. Finally, based on STRUCTURE, majority of Karen strains belonged to hpAsia2 population type. These results supported that Karen ancestry seems to be related with West Asia, a region with similar genotypes and population type as Karen strains [3]. Based on the history within Karen peoples, they arrived as refugee to Myanmar from North and are establishing themselves along the border between Thailand and Myanmar [50]. A mitochondrial DNA study showed that origin of Bamar as a predominant ethnic in Myanmar (68%) was different with Karen, even though they shared Tibeto-Burma origin and same source ancestor from Northwestern of China [51, 52].

Although Hmong contained strains with proportion of *vacA* m1 as similar as Thai, they had higher proportion of East-Asian-type *cagA* and *iceA1* positive strains. On the other hand, *vacA* m1 predominant was a differentiator with Thai-Chinese. Additionally, although most of Hmong strains belonged to hspEAsia population as same as Thai-Chinese, pre-EPIYA 18-bp deletion type was predominant. The 18-bp deletion type had been reported as the majority type in Vietnamese population [24]. Thus, there are two implications to explain these data. First, Hmong may become a high risk population in Thailand associated with higher prevalence and severer virulence genotypes of *H. pylori* (East-Asian-type *cagA* with 18-bp deletion). Second, Hmong ancestry source will be probably different from Thai or Thai-Chinese and assumed to have some connection with Vietnamese based on similarity of pre-EPIYA genotype. When we only compared *H. pylori* with East-Asian-type *cagA* and categorized onto hspEAsia from Bhutan, Hmong in Maesot (this study) and Hmong in Vietnam (unpublished data), they closed each other and make one group in the phylogenic tree (Additional file 5: Figure S4.), which indicated a possibility of similar origin. It is believed that second half of the 19th century, the large number of Hmong settlers migration from Sichuan, Guizhou and Yunnan, penetrated the Peninsula and went as far south as the 17th parallel near Tak in Thailand [53].

Our results confirmed our previous study [13] that Thai-Chinese strains contained East-Asian-type *cagA* and *vacA* m2 as the major genotypes. The *vacA* m2 genotype is common among ethnic Chinese in the Southern parts of East Asia [8, 28]. We completed our previous study with confirmation that *H. pylori* from Thai-Chinese ethnic contained pre-EPIYA 39-bp deletion type which is also predominantly in East Asian countries [24]. Additionally, STRUCTURE also confirmed the strains belonged to hspEAsia. The influences of Chinese in Thailand started from 27th century before present when people from Guangxi, China settled in Northeast Thailand. During Ayutthaya period (13th century) many Chinese intermarried with local Thai that while infusing of Chinese culture in Thailand and continuously until the late of 18th century which mostly from Cháozhōu prefecture [54]. The latest, they have immigrated to Thailand from South-West China (e.g. Yunnan) [13].

In contrast with our previous study [13] that majority of *cagA* genotype in strains isolated from Thai in Bangkok (capital city) was Western-type, Thai strains in Maesot contained East-Asian-type *cagA*. It is indicated that generally Thai in Maesot had higher risk for gastric cancer than Thai who living in the capital that may also partly explained the difference of gastric cancer risk between two regions (ASR female and male in North vs Central were 4.1, 5.4 vs. 1.8, 3.8, respectively) [55]. STRUCTURE showed that Thai strains belonged to three populations; hpAsia2, hpEurope and hspEAsia. hpAsia2 and hpEurope population may reflect cultural influences from India into Thailand including Buddhism (∼ 2300 years ago). India population also belonged to hpAsia2 and hpEurope [56], however the more recent importation also should be considered. Started 600 years ago Thailand contacted with European countries including Portugal, Spain, French, Denmark, Netherlands and England [57].

Previous study showed that α-EAS Ab could detect in the most of Japanese East-Asian-type *cagA* strains [17, 29]. In contrast, we found that only 71.4% of East-Asian-type CagA of Maesot strains could detect by same antibody due to an unidentical sequences for the α-EAS Ab designed amino acid sequence. These differences could explained with several reasons. First, previous study recruited 166 patients from center area of Thailand that 44 of them (83.0%) were Western-type CagA and only 9 (17.0%) were East-Asian type *cagA*. Thus, it may influenced the sensitivity rate of the test. Importantly, the kit showed a consistency to exclude all Western-type *cagA* in Thai strains in both of study. Second, Thailand is a cultural cross roads between East and South Asia which contained a lot of recombination genotype among strains including CagA. It is supported by the big diversity of CagA epitope in this study that could not detect by α-EAS Ab (3–14 amino acid differences). Therefore, the kit could not use in the entire of Thailand region and a validation in each region is a critical importance. Our group also reported a low immunoreactivity of α-EAS Ab in East-Asian-type CagA Bhutanese strains [58], suggesting that the α-EAS antibody might specific for each country.

Finally, we had some limitations in this study. Since we conducted a community-based endoscopic survey for only 2 days, we could not get the similar sample size of patients in each ethnic group. Although we could not find any difference in the prevalence/genotypes of virulence factors such as *babA, iceA, babA, jhp0562* and gastric mucosal status, further studies with larger number of patients, especially for Karen ethnic, will be necessary to confirm our current data.

## Conclusions

*H. pylori* genotypes were relatively different among the ethnic groups in Thailand and associated with the source of ancestor. Caution and check-up carefully are required on *H. pylori* infection in Hmong ethnic associated with high prevalence and severer virulence genotypes of *H. pylori*.

## Additional files

**Additional file 1: Table S1** Primers for detecting various virulence genes. **Table S2** Relationship between gastric mucosal status by upstream EPIYA sequences and ethnic group. **Table S3** Gastric mucosal status by *vacA* genotypes. **Table S4** The distribution of *H. pylori* population, *cagA *and *vacA* m genotypes and ethnicity

**Additional file 2.** Histological score according to genotype of *cagA* in Thai ethnic. The East-Asian-type *cagA* (n = 47) had significant higher of activity in antrum and body and atrophy and intestinal metaplasia in corpus than Western-type *cagA* genotype (n = 27, *P* < 0.05, Mann–Whitney U test)

**Additional file 3.** Histological score according to genotype of *cagA* in Karen ethnic. *H. pylori* harbored with East-Asian-type *cagA* (n = 4) induced activity and intestinal metaplasia higher than Western-type *cagA* (n = 11) genotype (*P* < 0.05, Mann–Whitney U test)

**Additional file 4.** Population of *H. pylori* Maesot strains by STRUCTURE. The *H. pylori* strains from 24 Thai, 20 Hmong, 15 Karen and 7 Thai-Chinese were analyzed by STRUCTURE with K from 8–15. Each horizontal bar represent one strain, and the color in a line are proportional to the probabilities that the strain belong to each population. Each color square represented the strain from Thai, Hmong, Karen and Thai-Chinese. The *H. pylori* Maesot strains were hspEAsia, hpAsia2 and hpEurope. Interestingly, *H. pylori* Hmong and Thai-Chinese strains were located in hspEAsia while *H. pylori* Karen strains were belonged to hpAsia2. Thai *H. pylori* strains were located in three populations

**Additional file 5.** Phylogenetic tree of *cagA* of hspEAsia strains from Bhutan, Hmong strains of Maesot and Hmong strains of Vietnam. We analysed 10 Bhutanese strains, 9 Hmong strains from Vietnam and 18 from Hmong Maesot with East-Asian-type *cagA* and hspEAsia. There was no different among *cagA* sequences among three groups

## Abbreviations

*H. pylori*: *Helicobacter pylori*
IHC: immunohistochemistry
ASR: age standardize incidence rate
PCR: polymerase chain reaction

## References

[1] Suerbaum S, Michetti P (2002). *Helicobacter pylori* infection. N Engl J Med.

[2] de Martel C, Forman D, Plummer M (2013). Gastric cancer: epidemiology and risk factors. Gastroenterol Clin North Am.

[3] Suzuki R, Shiota S, Yamaoka Y (2012). Molecular epidemiology, population genetics, and pathogenic role of *Helicobacter pylori*. Infect Genet Evol.

[4] Yamaoka Y (2010). Mechanisms of disease: *Helicobacter pylori* virulence factors. Nat Rev Gastroenterol Hepatol.

[5] Higashi H, Tsutsumi R, Fujita A, Yamazaki S, Asaka M, Azuma T, Hatakeyama M (2002). Biological activity of the *Helicobacter pylori* virulence factor CagA is determined by variation in the tyrosine phosphorylation sites. Proc Natl Acad Sci USA.

[6] Argent RH, Kidd M, Owen RJ, Thomas RJ, Limb MC, Atherton JC (2004). Determinants and consequences of different levels of CagA phosphorylation for clinical isolates of *Helicobacter pylori*. Gastroenterology.

[7] Miftahussurur M, Yamaoka Y, Graham DY (2017). *Helicobacter pylori* as an oncogenic pathogen, revisited. Expert Rev Mol Med.

[8] Yamaoka Y, Orito E, Mizokami M, Gutierrez O, Saitou N, Kodama T, Osato MS, Kim JG, Ramirez FC, Mahachai V, Graham DY (2002). *Helicobacter pylori* in North and South America before Columbus. FEBS Lett.

[9] Shiota S, Suzuki R, Yamaoka Y (2013). The significance of virulence factors in *Helicobacter pylori*. J Dig Dis.

[10] Wirth T, Wang X, Linz B, Novick RP, Lum JK, Blaser M, Morelli G, Falush D, Achtman M (2004). Distinguishing human ethnic groups by means of sequences from *Helicobacter pylori*: lessons from Ladakh. Proc Natl Acad Sci USA.

[11] Linz B, Balloux F, Moodley Y, Manica A, Liu H, Roumagnac P, Falush D, Stamer C, Prugnolle F, van der Merwe SW (2007). An African origin for the intimate association between humans and *Helicobacter pylori*. Nature.

[12] Falush D, Wirth T, Linz B, Pritchard J, Stephens M, Kidd M, Blaser M, Graham D, Vacher S, Perez-Perez G (2003). Traces of human migrations in *Helicobacter pylori* populations. Science.

[13] Vilaichone RK, Mahachai V, Tumwasorn S, Wu JY, Graham DY, Yamaoka Y (2004). Molecular epidemiology and outcome of *Helicobacter pylori* infection in Thailand: a cultural cross roads. Helicobacter.

[14] Dixon M, Genta R, Yardley J, Correa P (1996). Classification and grading of gastritis. The updated Sydney System. International Workshop on the Histopathology of Gastritis, Houston 1994. Am J Surg Pathol.

[15] Bornschein J, Selgrad M, Wex T, Kuester D, Malfertheiner P (2012). Serological assessment of gastric mucosal atrophy in gastric cancer. BMC Gastroenterol.

[16] Rugge M, Meggio A, Pennelli G, Piscioli F, Giacomelli L, De Pretis G, Graham DY (2007). Gastritis staging in clinical practice: the OLGA staging system. Gut.

[17] Kanada R, Uchida T, Tsukamoto Y, Nguyen LT, Hijiya N, Matsuura K, Kodama M, Okimoto T, Murakami K, Fujioka T (2008). Genotyping of the *cagA* gene of *Helicobacter pylori* on immunohistochemistry with East Asian cagA-specific antibody. Pathol Int.

[18] Vilaichone RK, Panarat W, Aekpongpaisit S, Mahachai V (2014). Clinical Characteristics and *Helicobacter pylori* status of gastric cancer in Thailand. Asian Pac J Cancer Prev.

[19] Nguyen LT, Uchida T, Kuroda A, Tsukamoto Y, Trinh TD, Ta L, Mai HB, Ho DQ, Hoang HH, Vilaichone RK (2009). Evaluation of the anti-East Asian cagA-specific antibody for cagA phenotyping. Clin Vaccine Immunol.

[20] Yamaoka Y, El-Zimaity H, Gutierrez O, Figura N, Kim J, Kodama T, Kashima K, Graham D, Kim J (1999). Relationship between the cagA 3′ repeat region of *Helicobacter pylori*, gastric histology, and susceptibility to low pH. Gastroenterology.

[21] Atherton J, Cao P, Peek RJ, Tummuru M, Blaser M, Cover T (1995). Mosaicism in vacuolating cytotoxin alleles of *Helicobacter pylori*. Association of specific *vacA* types with cytotoxin production and peptic ulceration. J Biol Chem.

[22] Yamazaki S, Yamakawa A, Okuda T, Ohtani M, Suto H, Ito Y, Yamazaki Y, Keida Y, Higashi H, Hatakeyama M, Azuma T (2005). Distinct diversity of *vacA*, *cagA*, and *cagE* genes of *Helicobacter pylori* associated with peptic ulcer in Japan. J Clin Microbiol.

[23] Matsunari O, Shiota S, Suzuki R, Watada M, Kinjo N, Murakami K, Fujioka T, Kinjo F, Yamaoka Y (2012). Association between *Helicobacter pylori* virulence factors and gastroduodenal diseases in Okinawa, Japan. J Clin Microbiol.

[24] Uchida T, Nguyen LT, Takayama A, Okimoto T, Kodama M, Murakami K, Matsuhisa T, Trinh TD, Ta L, Ho DQ (2009). Analysis of virulence factors of *Helicobacter pylori* isolated from a Vietnamese population. BMC Microbiol.

[25] Rhead JL, Letley DP, Mohammadi M, Hussein N, Mohagheghi MA, Eshagh Hosseini M, Atherton JC (2007). A new *Helicobacter pylori* vacuolating cytotoxin determinant, the intermediate region, is associated with gastric cancer. Gastroenterology.

[26] Basiri Z, Safaralizadeh R, Bonyadi MJ, Somi MH, Mahdavi M, Latifi-Navid S (2014). *Helicobacter pylori vacA* d1 genotype predicts risk of gastric adenocarcinoma and peptic ulcers in northwestern Iran. Asian Pac J Cancer Prev.

[27] Bakhti SZ, Latifi-Navid S, Mohammadi S, Zahri S, Bakhti FS, Feizi F, Yazdanbod A, Siavoshi F. Relevance of *Helicobacter pylori vacA* 3′-end region polymorphism to gastric cancer. Helicobacter. 2015;21(4):305–16. 10.1111/hel.12284 26612250

[28] Mukhopadhyay AK, Kersulyte D, Jeong JY, Datta S, Ito Y, Chowdhury A, Chowdhury S, Santra A, Bhattacharya SK, Azuma T (2000). Distinctiveness of genotypes of *Helicobacter pylori* in Calcutta, India. J Bacteriol.

[29] Uchida T, Kanada R, Tsukamoto Y, Hijiya N, Matsuura K, Yano S, Yokoyama S, Kishida T, Kodama M, Murakami K (2007). Immunohistochemical diagnosis of the *cagA*-gene genotype of *Helicobacter pylori* with anti-East Asian CagA-specific antibody. Cancer Sci.

[30] Perez-Perez GI, Taylor DN, Bodhidatta L, Wongsrichanalai J, Baze WB, Dunn BE, Echeverria PD, Blaser MJ (1990). Seroprevalence of *Helicobacter pylori* infections in Thailand. J Infect Dis.

[31] Chinprasatsak S, Wilairatana P, Visalwadi P, Sanguansri P, Batara L, Kityaporn D, Looareesuwan S, Kurathong S, Charoenlarp P (1993). *Helicobacter pylori* prevalence in northeastern Thailand. Southeast Asian J Trop Med Public Health.

[32] Isenbarger DW, Bodhidatta L, Hoge CW, Nirdnoy W, Pitarangsi C, Umpawasiri U, Echeverria P (1998). Prospective study of the incidence of diarrheal disease and *Helicobacter pylori* infection among children in an orphanage in Thailand. Am J Trop Med Hyg.

[33] Sriamporn S, Setiawan V, Pisani P, Suwanrungruang K, Sirijaichingkul S, Mairiang P, Parkin DM (2002). Gastric cancer: the roles of diet, alcohol drinking, smoking and *Helicobacter pylori* in northeastern Thailand. Asian Pac J Cancer Prev.

[34] Tangmankongworakoon N, Mahachai V, Thong-Ngam D, Vilaichone RK, Tumwasorn S, Kullavanijaya P (2003). Pattern of drug resistant *Helicobacter pylori* in dyspeptic patients in Thailand. J Med Assoc Thai.

[35] Atisook K, Kachinthorn U, Luengrojanakul P, Tanwandee T, Pakdirat P, Puapairoj A (2003). Histology of gastritis and *Helicobacter pylori* infection in Thailand: a nationwide study of 3776 cases. Helicobacter.

[36] Mitipat N, Siripermpool P, Jadwattanakul T, Chaunthongkum S (2005). The prevalence of *Helicobacter pylori* infection in patients with gastrointestinal symptoms in Chon Buri, Thailand. Southeast Asian J Trop Med Public Health.

[37] Linpisarn S, Suwan W, Lertprasertsuk N, Koosirirat C, Steger HF, Prommuangyong K, Phornphutkul K (2007). *Helicobacter pylori cagA*, *vacA* and *iceA* genotypes in northern Thai patients with gastric disease. Southeast Asian J Trop Med Public Health.

[38] Chomvarin C, Namwat W, Chaicumpar K, Mairiang P, Sangchan A, Sripa B, Tor-Udom S, Vilaichone RK (2008). Prevalence of *Helicobacter pylori vacA*, *cagA*, *cagE*, *iceA* and *babA2* genotypes in Thai dyspeptic patients. Int J Infect Dis.

[39] Tanuma M, Rimbara E, Noguchi N, Boonyaritichaikij S, Kuwabara K, Fukunaga Y, Sasatsu M (2009). Analysis of clarithromycin resistance and cagA status in *Helicobacter pylori* by use of feces from children in Thailand. J Clin Microbiol.

[40] Hirai I, Sasaki T, Kimoto A, Yamamoto Y, Azuma T, Mahachai V, Hansomburana P, Lertkupinit C, Luangjaru S, Noophan P (2010). Infection of less virulent *Helicobacter pylori* strains in asymptomatic healthy individuals in Thailand as a potential contributing factor to the Asian enigma. Microbes Infect.

[41] Vilaichone RK, Mahacahai V, Tumwasorn S, Kachintorn U (2011). *CagA* genotype and metronidazole resistant strain of *Helicobacter pylori* in functional dyspepsia in Thailand. J Gastroenterol Hepatol.

[42] Chomvarin C, Phusri K, Sawadpanich K, Mairiang P, Namwat W, Wongkham C, Hahnvajanawong C (2012). Prevalence of cagA EPIYA motifs in *Helicobacter pylori* among dyspeptic patients in northeast Thailand. Southeast Asian J Trop Med Public Health.

[43] Sakonlaya D, Apisarnthanarak A, Yamada N, Tomtitchong P (2014). Modified toluidine blue: an alternative stain for *Helicobacter pylori* detection in routine diagnostic use and post-eradication confirmation for gastric cancer prevention. Asian Pac J Cancer Prev.

[44] Piriyapong K, Tangaroonsanti A, Mahachai V, Vilaichone RK (2014). *Helicobacter pylori* infection impacts on functional dyspepsia in Thailand. Asian Pac J Cancer Prev.

[45] Uchida T, Miftahussurur M, Pittayanon R, Vilaichone RK, Wisedopas N, Ratanachu-Ek T, Kishida T, Moriyama M, Yamaoka Y, Mahachai V (2015). *Helicobacter pylori* infection in Thailand: a nationwide study of the cagA phenotype. PLoS ONE.

[46] Higashi H, Tsutsumi R, Muto S, Sugiyama T, Azuma T, Asaka M, Hatakeyama M (2002). SHP-2 tyrosine phosphatase as an intracellular target of *Helicobacter pylori* CagA protein. Science.

[47] Hatakeyama M, Higashi H (2005). *Helicobacter pylori* CagA: a new paradigm for bacterial carcinogenesis. Cancer Sci.

[48] Hatakeyama M (2004). Oncogenic mechanisms of the *Helicobacter pylori* CagA protein. Nat Rev Cancer.

[49] Miftahussurur M, Syam AF, Makmun D, Nusi IA, Zein LH, Zulkhairi Akil F, Uswan WB, Simanjuntak D, Uchida T (2015). *Helicobacter pylori* virulence genes in the five largest islands of Indonesia. Gut Pathog.

[50] Moonieinda V. The Karen people: culture, faith and history. The Karen Buddhist Dhamma Dhutta Foundation. 2011.

[51] Summerer M, Horst J, Erhart G, Weißensteiner H, Schönherr S, Pacher D, Forer L, Horst D, Manhart A, Horst B (2014). Large-scale mitochondrial DNA analysis in Southeast Asia reveals evolutionary effects of cultural isolation in the multi-ethnic population of Myanmar. BMC Evol Biol.

[52] Wen B, Xie X, Gao S, Li H, Shi H, Song X, Qian T, Xiao C, Jin J, Su B (2004). Analyses of genetic structure of Tibeto-Burman populations reveals sex-biased admixture in southern Tibeto-Burmans. Am J Hum Genet.

[53] Michaud J (1997). From southwest China into upper Indochina: an overview of Hmong (Miao) migrations. Asia Pac Viewp.

[54] Kutanan W, Srikummool M, Pittayaporn P, Seielstad M, Kangwanpong D, Kumar V, Prombanchachai T, Chantawannakul P (2015). Admixed origin of the Kayah (Red Karen) in northern Thailand revealed by biparental and paternal markers. Ann Hum Genet.

[55] Imsamran W, Chaiwerawattana A, Wiangnon S, Pongnikorn D, Suwanrungrung K, Sangrajrang S, Buasom R (2015). Cancer in Thailand.

[56] Devi SM, Ahmed I, Francalacci P, Hussain MA, Akhter Y, Alvi A, Sechi LA, Megraud F, Ahmed N (2007). Ancestral European roots of *Helicobacter pylori* in India. BMC Genomics.

[57] Rajanubhab D. The introduction of Western culture in Siam. 1952:89–100. (pp. 89–100).

[58] Matsunari O, Miftahussurur M, Shiota S, Suzuki R, Vilaichone RK, Uchida T, Ratanachu-ek T, Tshering L, Mahachai V, Yamaoka Y (2016). Rare *Helicobacter pylori* virulence genotypes in Bhutan. Sci Rep.

