# Supplementary material for: Helicobacter pylori virulence genes of minor ethnic groups in North Thailand
Source: Gut Pathog. 2017 Oct 11;9:56. doi: 10.1186/s13099-017-0205-x (PMC5637267; doi:10.1186/s13099-017-0205-x)
Supplement: Supplementary file 1 — Additional file 1: Table S1 Primers for detecting various virulence genes. Table S2 Relationship between gastric mucosal status by upstream EPIYA sequences and ethnic group. Table S3 Gastric mucosal status by vacA genotypes. Table S4 The distribution of H. pylori population, cagA and vacA m genotypes and ethnicity [file 13099_2017_205_MOESM1_ESM.doc]

**Table S1. Primer for detecting various virulence genes**

| Gene | Primer 5’-3’ | Product size (bp) |
| --- | --- | --- |
| *vacA*  *vacA* s region  *vacA* m region  *vacA* i region  *vacA* d region  *vacA* c region  c1 allele  c2 allele | VA1F: ATG GAA ATA CAA CAA ACA CAC  VA1R: CTG CTT GAA TGC GCC AAA C  VAGF: CAA TCT GTC CAA TCA AGC GAG  VAGR: GCG TCT AAA TAA TTC CAA GG  VacF1: GTT GGG ATT GGG GGA ATG CCG  C1R: TTA ATT TAACGC TGT TTG AAG  C2R: GAT CAA CGC TCT GAT TTG A  VAS5F: ACT AAT ATT GGC ACA CTG GAT TTG  VAGFR: CTC GCT TGA TTG GAC AGA TTG  C1F: ATC ATY SGT TAT GRH AAT GTT TCT  Rnd: TTA TGC TCT AAA CTG GCT A  C2F: ATT ATA ATT TAG GAG TGC AAG G  Rnd: TTA TGC TCT AAA CTG GCT A | 259-286  567-642  i1 426  i2 432  d1 367-379  d2 298  c1 600-700  c2 600-700 |
| *cagA* | OMF: AGC AAA AAG CGA CCT TGA AA  OMR: ATT CAC GAG CTT CAG CCA CT | 521 |
| *dupA* | dupAF2: ATG TTT CTT GGT TTA GAG GG  dupAR2: TTA TAC ATA TTG AAT AAT CTC GC | 2499 |
| *babA* | babAF: CCA AAC GAA ACA AAA AGC GT  babAR: GCT TGT GTA AAA GCC GTG GT | 271 |
| *iceA*  *iceA*1  *iceA*2 | iceA1F: GTG TTT TTA ACC AAA GTA TC  iceA1R: CTA TAG CCA STY TCT TTG CA  iceA2F: GTT GGG TAT ATC ACA ATT TAT  iceA2R: TTT CCC TAT TTT CTA GTA GGT | 247  229 |
| *jhp0562/β-(1,3)gal T* | jhp0562F: TGA AAA GCC CTT TTG ATT TTG  jhp0562R: GCT GTA GTG GCC ACA TAC ACG | 301/602 |

**Table S2. Relationship between gastric mucosal status by upstream EPIYA sequences and ethnic group**

| Ethnics | Type of delete |  | Antrum (mean[median]) | | | | | Corpus (mean[median]) | | | | | |
| --- | --- | --- | --- | --- | --- | --- | --- | --- | --- | --- | --- | --- | --- |
|  |  | N | Activity | Inflammation | Atrophy | Intestinal  metaplasia | HP  density | Activity | Inflammation | Atrophy | Intestinal metaplasia | HP  density | OLGA  (mean [median]) |
| Total | Non delete | 44 | 1.3 [1]^b^ | 1.6 [2] | 1.1 [1] | 0.0 [0]^b^ | 1.6 [2] | 0.7 [1]^b^ | 0.8 [1] | 0.1 [0] | 0.0 [0] | 1.5 [1] | 1.1 [1] |
|  | 18 bp | 58 | 1.6 [2]^b^ | 1.8 [2] | 1.3 [1] | 0.3 [0]^b^ | 1.7 [2] | 1.1 [1]^b^ | 1.0 [1] | 0.2 [0] | 0.0 [0] | 1.7 [2] | 1.3 [1] |
|  | 39 bp | 44 | 1.5 [2] | 1.8 [2] | 1.2 [1] | 0.2 [1] | 1.5 [1] | 0.8 [1]^b^ | 0.8 [1] | 0.2 [0] | 0.0 [0] | 1.4 [1] | 1.2 [1] |
| Thai | Non delete | 30 | 1.3 [1]^a^ | 1.6 [2] | 1.0 [1] | 0.0 [0]^c^ | 1.6 [2] | 0.8 [1]^c^ | 0.9 [1] | 0.2 [0] | 0.1 [0] | 1.5 [1] | 1.0 [1]^a^ |
|  | 18 bp | 25 | 1.8 [2]^a,c^ | 1.9 [2] | 1.5 [2]^c^ | 0.2 [0] | 1.7 [2] | 1.2 [1]^c,b^ | 1.0 [1] | 0.2 [0] | 0.0 [0] | 1.7 [2] | 1.5 [2]^a, c^ |
|  | 39 bp | 19 | 1.4 [1]^c^ | 1.6 [2] | 1.2 [1]^c^ | 0.3 [0]^c^ | 1.5 [1] | 0.7 [1]^b^ | 0.8 [1] | 0.2 [0] | 0.0 [0] | 1.5 [1] | 1.2 [1]^c^ |
| Hmong | Non delete | 2 | 2 [2] | 2 [2] | 2 [2] | 0.0 [0] | 2 [2] | 0.0 [0] | 0.0 [0] | 0.0 [0] | 0.0 [0] | 1 [1] | 2 [2] |
|  | 18 bp | 27 | 1.3 [1] | 1.7 [2] | 1.1 [1] | 0.3 [0] | 1.6 [2] | 1.0 [1] | 1.0 [1] | 0.1 [0] | 0.0 [0] | 1.6 [2] | 1.1 [1] |
|  | 39 bp | 21 | 1.7 [2] | 2.0 [2] | 1.3 [1] | 0.0 [0] | 1.6 [1] | 0.9 [1] | 0.9 [1] | 0.2 [0] | 0.0 [0] | 1.4 [1] | 1.3 [1] |
| Karen | Non delete | 11 | 1.4 [1]^c^ | 1.5 [1] | 1.4 [1] | 0.0 [0]^c^ | 1.5 [2] | 0.6 [1] | 0.5 [0] | 0.1 [0] | 0.0 [0] | 1.6 [1] | 1.4 [1] |
|  | 18 bp | 4 | 2.3 [2]^c^ | 1.8 [2] | 1.3 [1] | 0.8 [1]^c^ | 1.5 [2] | 1.3 [1] | 1.0 [1] | 0.3 [0] | 0.0 [0] | 1.3 [1] | 1.3 [1] |
|  | 39 bp | 0 | 0.0 [0] | 0.0 [0] | 0.0 [0] | 0.0 [0] | 0.0 [0] | 0.0 [0] | 0.0 [0] | 0.0 [0] | 0.0 [0] | 0.0 [0] | 0.0 [0] |
| Thai-  Chinese | Non delete | 1 | 2 [2] | 1 [1] | 1 [1] | 0 [0] | 1 [1] | 1 [1] | 1 [1] | 0 [0] | 0 [0] | 1 [1] | 1 [1] |
|  | 18 bp | 2 | 2 [2] | 2 [2] | 1.5 [2] | 0 [0] | 2 [2] | 2 [2] | 1.5 [2] | 0.5 [1] | 0 [0] | 2.5 [3] | 1.5 [2] |
|  | 39 bp | 4 | 1.0 [1] | 1.5 [2] | 0.8 [1] | 0.3 [0] | 1.5 [2] | 0.8 [1] | 1.0 [1] | 0.3 [0] | 0.0 [0] | 1.3 [2] | 0.8 [1] |

a, P< 0.001; b, P≤ 0.01; c, P≤ 0.05

**Table S3. Gastric mucosal status by *vacA* genotype**

| *vacA* genotype |  | Antrum (mean[median]) | | | | | Corpus (mean[median]) | | | | |  |
| --- | --- | --- | --- | --- | --- | --- | --- | --- | --- | --- | --- | --- |
|  | N | Activity | Inflammation | Atrophy | Intestinal  metaplasia | HP  density | Activity | Inflammation | Atrophy | Intestinal metaplasia | HP  density | OLGA  (mean [median]) |
| s1 | 151 | 1.5 [2] | 1.72 [2] | 1.21 [1] | 0.16 [0] | 1.58 [2] | 0.87 [1] | 0.86 [1] | 0.16 [0] | 0.01 [0] | 1.53 [1] | 1.23 [1] |
| s2 | 1 | 1.0 [0] | 1.0 [0] | 1.0 [0] | 0.0 [0] | 1.0 [0] | 0.0 [0] | 0.0 [0] | 0.0 [0] | 0.0 [0] | 1.0 [0] | 1.0 [0] |
| m1 | 91 | 1.47 [2] | 1.77 [2] | 1.22 [1] | 0.18 [0] | 1.52 [1] | 0.93 [1] | 0.85 [1] | 0.19 [0] | 0.0 [0] | 1.53 [1] | 1.23 [1] |
| m2 | 57 | 1.54 [2] | 1.65 [2] | 1.21 [1] | 0.14 [0] | 1.72 [2] | 0.79 [1] | 0.88 [1] | 0.12 [0] | 0.04 [0] | 1.54 [1] | 1.23 [1] |
| m1 and 2 | 4 | 1.25 [1.5] | 1.5 [2] | 1.0 [1] | 0.0 [0] | 1.0 [1] | 0.5 [0.5] | 0.75 [1] | 0.0 [0] | 0.0 [0] | 1.25 [1.5] | 1.0 [1] |
| i1 | 148 | 1.49 [2] | 1.74 [2] | 1.21 [1] | 0.16 [0] | 1.59 [2] | 0.88 [1] | 0.86 [1] | 0.16 [0] | 0.1 [0] | 1.53 [1] | 1.22 [1] |
| i2 | 2 | 1.5 [1.5] | 1.0 [1] | 1.0 [1] | 0.0 [0] | 1.0 [1] | 0.5 [0.5] | 0.5 [0.5] | 0.0 [0] | 0.0 [0] | 1.0 [1] | 1.0 [1] |
| negative | 2 | 1.5 [1.5] | 1.0 [1] | 1.5 [1.5] | 0.0 [0] | 1.5 [1.5] | 0.5 [0.5] | 0.5 [0.5] | 0.5 [0.5] | 0.0 [0] | 1.5 [1.5] | 1.5 [1.5] |
| d1 | 148 | 1.49 [2] | 1.73 [2] | 1.21 [1] | 0.16 [0] | 1.59 [2] | 0.89 [1] | 0.87 [1] | 0.16 [0] | 0.01 [0] | 1.54 [1] | 1.22 [1] |
| d2 | 3 | 1.33 [1] | 1.33 [1] | 1.0 [1] | 0.0 [0] | 1.0 [1] | 0.33 [0] | 0.33 [0] | 0.0 [0] | 0.0 [0] | 1.0 [1] | 1.0 [1] |
| negative | 1 | 2.0 [2] | 1.0 [1] | 2.0 [2] | 0.0 [0] | 2.0 [2] | 0.0 [0] | 0.0 [0] | 0.0 [0] | 0.0 [0] | 1.0 [1] | 2.0 [2] |
| c1 | 80 | 1.46 [1.5] | 1.73 [2] | 1.19 [1] | 0.16 [0] | 1.53[1] | 0.94 [1] | 0.83 [1] | 0.19 [0] | 0.0 [0] | 1.56 [1] | 1.2 [1] |
| c2 | 37 | 1.46 [2] | 1.73 [2] | 1.22 [1] | 0.14 [0] | 1.54 [1] | 0.81 [1] | 0.97 [1] | 0.11 [0] | 0.0 [0] | 1.43 [1] | 1.2 [1] |
| c1 and c2 | 30 | 1.6 [2] | 1.7 [2] | 1.2 [1] | 0.2 [0] | 1.7 [2] | 0.83 [1] | 0.87 [1] | 0.17 [0] | 0.07 [0] | 1.63 [2] | 1.23 [1] |
| negative | 5 | 1.6 [2] | 1.6 [2] | 1.6 [2] | 0.0 [0] | 0.0 [0] | 2.0 [2] | 0.40 [0] | 0.0 [0] | 0.0 [0] | 1.0 [1] | 1.6 [2] |

**Table S4. The distribution of *H. pylori* population*, cagA* and *vacA* m genotype and by ethnicity**

|  | hspEAsia | | | | | | | | hpAsia2 | | | | |
| --- | --- | --- | --- | --- | --- | --- | --- | --- | --- | --- | --- | --- | --- |
| Ethnic | East-Asian-type *cagA*  (n = 45) | | | Western-type *cagA*  (n = 2) | | *cagA* negative  (n = 3) | | Total | East-Asian-type *cagA*  (n = 3) | | Western-type *cagA*  (n = 25) | | Total |
|  | m1 | m2 | m1 and m2 | m1 | m2 | m1 | m2 |  | m1 | m2 | m1 | m2 |  |
| Total | 24  (48.0) | 20  (40.0) | 1  (2.0) | 1  (2.0) | 1  (2.0) | 1  (2.0) | 2  (4.0) | 50 | 2  (7.1) | 1  (3.6) | 24  (85.7) | 1  (3.6) | 28 |
| Thai | 10  (52.6) | 7  (36.8) | 1  (5.3) | 0  (0.0) | 1  (5.3) | 0  (0.0) | 0  (0.0) | 19 | 0  (0.0) | 0  (0.0) | 12  (92.3) | 1  (7.7) | 13 |
| Hmong | 12  (52.2) | 8  (34.8) | 0  (0.0) | 0  (0.0) | 0  (0.0) | 1  (4.3) | 2  (8.7) | 23 | 0  (0.0) | 0  (0.0) | 2  (100.0) | 0  (0.0) | 2 |
| Karen | 0  (0.0) | 1  (50.0) | 0  (0.0) | 1  (50.0) | 0  (0.0) | 0  (0.0) | 0  (0.0) | 2 | 2  (15.4) | 1  (7.7) | 10  (76.9) | 0  (0.0) | 13 |
| Thai-Chinese | 2  (33.3) | 4  (66.7) | 0  (0.0) | 0  (0.0) | 0  (0.0) | 0  (0.0) | 0  (0.0) | 6 | 0  (0.0) | 0  (0.0) | 0  (0.0) | 0  (0.0) | 0 |
