# Supplementary figures and images for: Helicobacter pylori virulence genes of minor ethnic groups in North Thailand
Source: Gut Pathog. 2017 Oct 11;9:56. doi: 10.1186/s13099-017-0205-x (PMC5637267; doi:10.1186/s13099-017-0205-x)

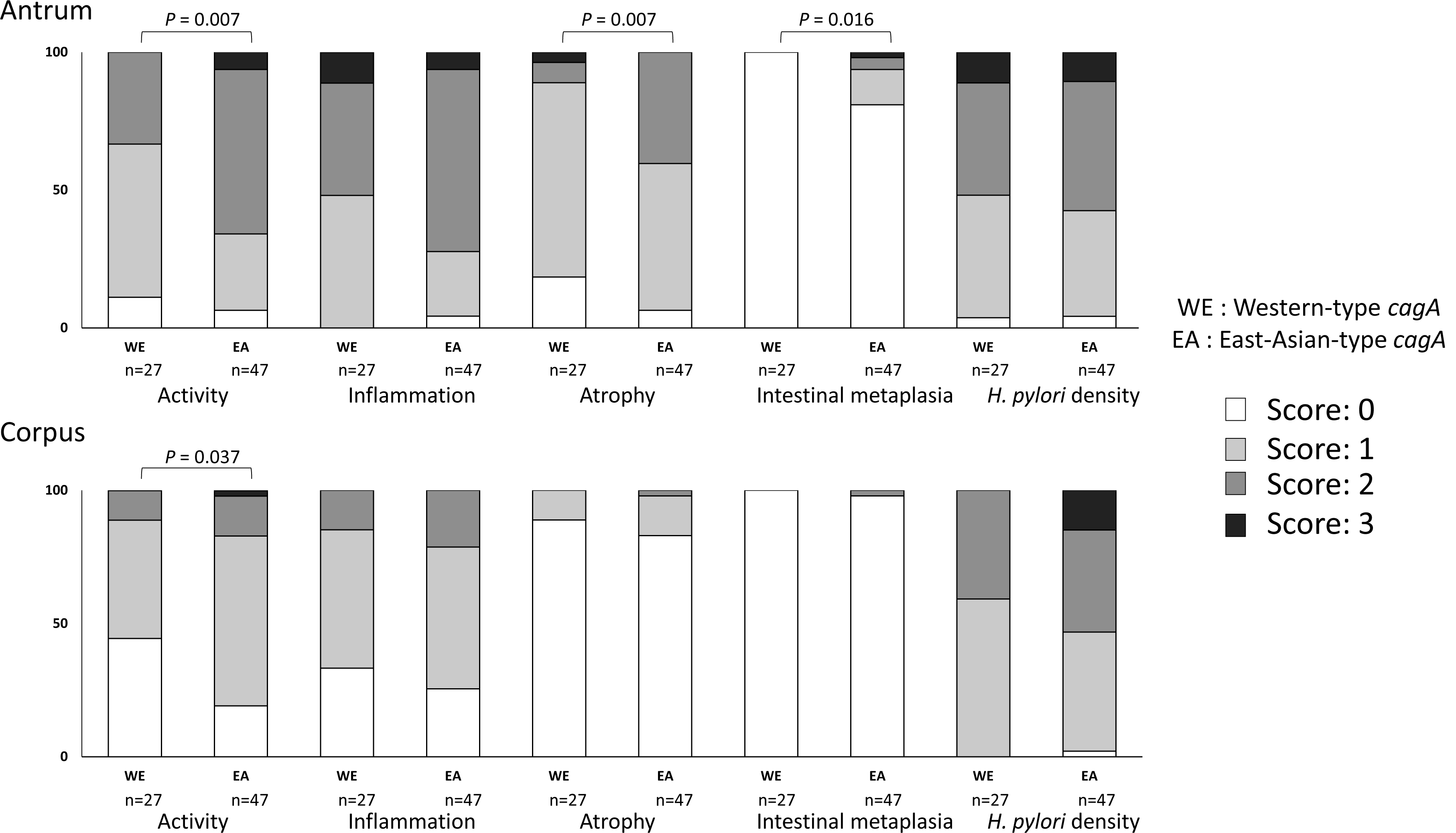

Supplement: Supplementary file 2 — Additional file 2. Histological score according to genotype of cagA in Thai ethnic. The East-Asian-type cagA (n = 47) had significant higher of activity in antrum and body and atrophy and intestinal metaplasia in corpus than Western-type cagA genotype (n = 27, P < 0.05, Mann–Whitney U test) [file 13099_2017_205_MOESM2_ESM.tif]

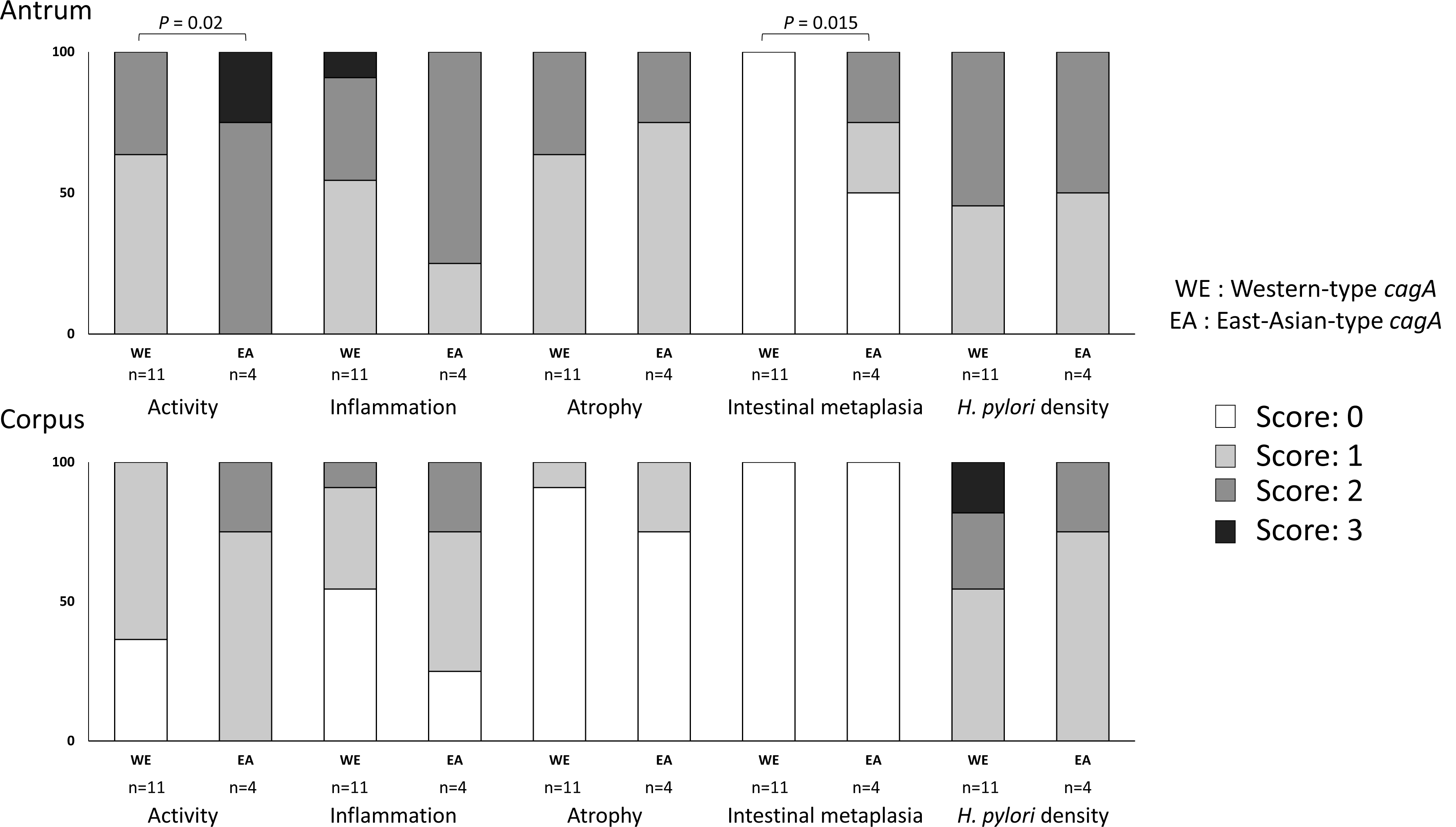

Supplement: Supplementary file 3 — Additional file 3. Histological score according to genotype of cagA in Karen ethnic. H. pylori harbored with East-Asian-type cagA (n = 4) induced activity and intestinal metaplasia higher than Western-type cagA (n = 11) genotype (P < 0.05, Mann–Whitney U test) [file 13099_2017_205_MOESM3_ESM.tif]

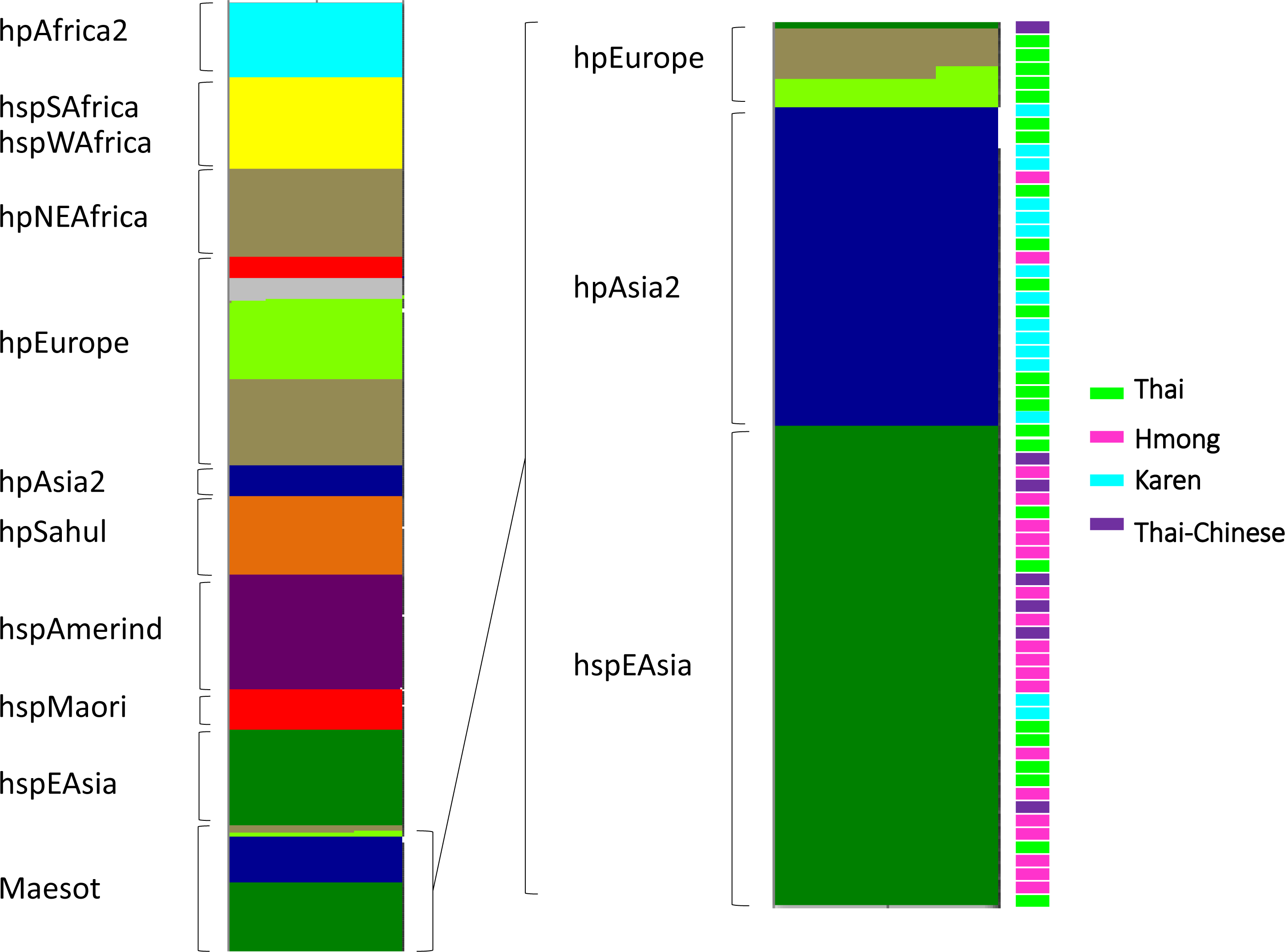

Supplement: Supplementary file 4 — Additional file 4. Population of H. pylori Maesot strains by STRUCTURE. The H. pylori strains from 24 Thai, 20 Hmong, 15 Karen and 7 Thai-Chinese were analyzed by STRUCTURE with K from 8–15. Each horizontal bar represent one strain, and the color in a line are proportional to the probabilities that the strain belong to each population. Each color square represented the strain from Thai, Hmong, Karen and Thai-Chinese. The H. pylori Maesot strains were hspEAsia, hpAsia2 and hpEurope. Interestingly, H. pylori Hmong and Thai-Chinese strains were located in hspEAsia while H. pylori Karen strains were belonged to hpAsia2. Thai H. pylori strains were located in three populations [file 13099_2017_205_MOESM4_ESM.tif]

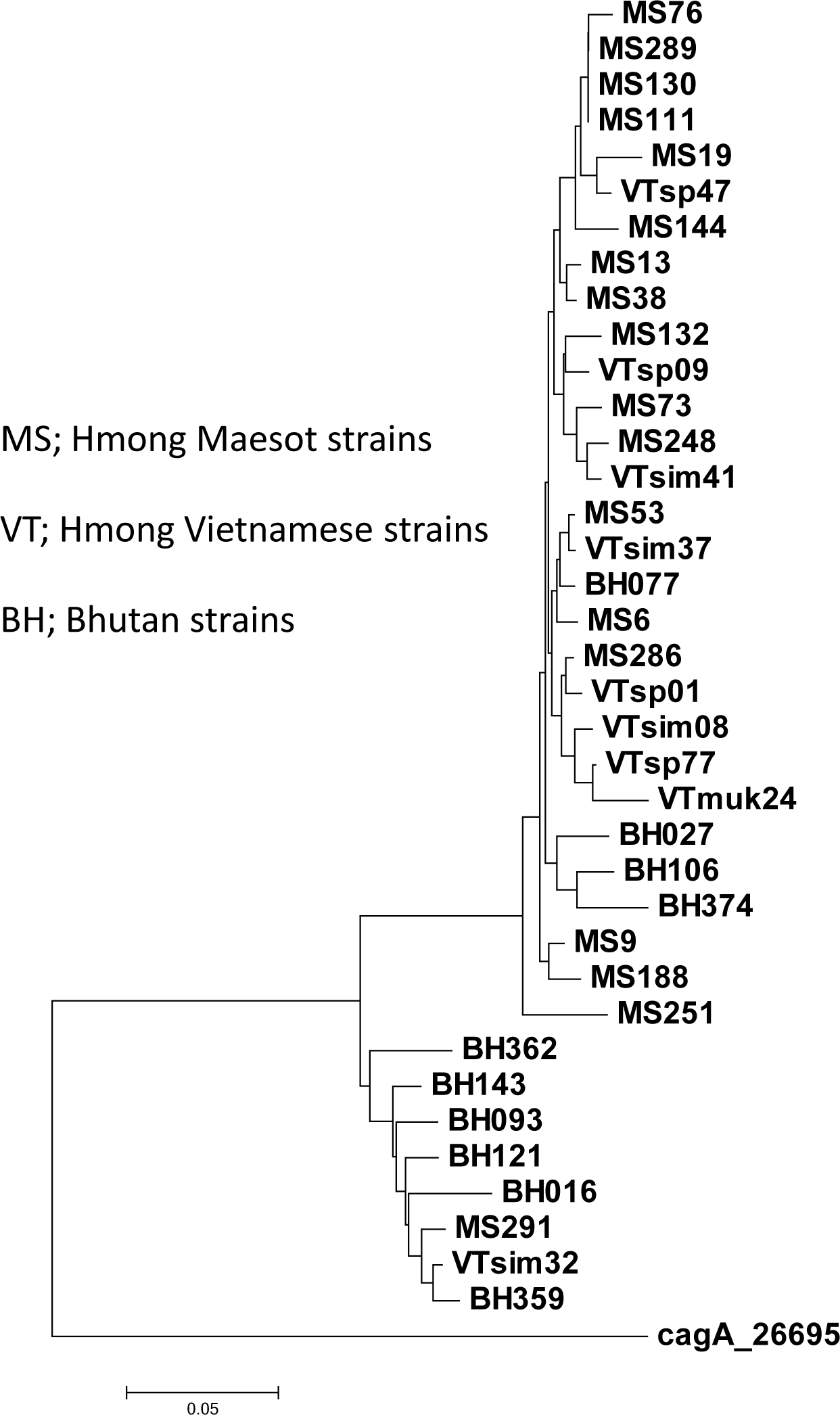

Supplement: Supplementary file 5 — Additional file 5. Phylogenetic tree of cagA of hspEAsia strains from Bhutan, Hmong strains of Maesot and Hmong strains of Vietnam. We analysed 10 Bhutanese strains, 9 Hmong strains from Vietnam and 18 from Hmong Maesot with East-Asian-type cagA and hspEAsia. There was no different among cagA sequences among three groups [file 13099_2017_205_MOESM5_ESM.tif]
